# Isolation, Characterization, and Transcriptome Analysis of an ISKNV-Like Virus from Largemouth Bass

**DOI:** 10.3390/v15020398

**Published:** 2023-01-30

**Authors:** Zhuqing Xu, Jiaming Liao, Dongzhuo Zhang, Shaoli Liu, Luhao Zhang, Shaozhu Kang, Linting Xu, Hong Chen, Wenquan Peng, Sheng Zhou, Qiwei Qin, Jingguang Wei

**Affiliations:** 1 Guangdong Laboratory for Lingnan Modern Agriculture, College of Marine Sciences, South China Agricultural University, Guangzhou 510642, China; 2 Guangdong Winsun Biological Pharmaceutical Co., Ltd., Guangzhou 511356, China; 3 Laboratory for Marine Biology and Biotechnology, Pilot National Laboratory for Marine Science and Technology (Qingdao), Qingdao 266000, China; 4 Department of Biological Sciences, National University of Singapore, Singapore 117543, Singapore

**Keywords:** *Micropterus salmoides*, ISKNV-ZY, isolation, characterization, transcriptome

## Abstract

Largemouth bass (*Micropterus salmoides*) is an important commercial fish farmed in China. Challenges related to diseases caused by pathogens, such as iridovirus, have become increasingly serious. In 2017, we detected iridovirus-infected diseased largemouth bass in Zunyi, Guizhou Province. The isolated virus was identified as an infectious spleen and kidney necrosis virus (ISKNV)-like virus (ISKNV-ZY). ISKNV-ZY induces a cytopathic effect after infecting mandarin fish brain (MFB) cells. Abundant hexagonal virus particles were observed in the cytoplasm of ISKNV-ZY-infected MFB cells, using electron microscopy. The whole genome of ISKNV-ZY contained 112,248 bp and 122 open reading frames. Phylogenetic tree analysis showed that ISKNV-ZY was most closely related to BCIV, indicating that it is an ISKNV-like megalocytivirus. ISKNV-ZY-infected largemouth bass started to die on day six and reached a death peak on days 7–8. Cumulative mortality reached 100% on day 10. Using RNA sequencing-based transcriptome analysis after ISKNV-ZY infection, 6254 differentially expressed unigenes (DEGs) were identified, of which 3518 were upregulated and 2673 downregulated. The DEGs were associated with endocytosis, thermogenesis, oxidative phosphorylation, the JAK-STAT signaling pathway, the MAPK signaling pathway, etc. These results contribute to understanding the molecular regulation mechanism of ISKNV infection and provide a basis for ISKNV prevention.

## 1. Introduction

Iridoviruses are a group of icosahedral cytoplasmic DNA viruses that can infect invertebrates and poikilothermic vertebrates, including insects, fish, amphibians, and reptiles [1]. At present, the family *Iridoviridae* contains seven genera, namely *Lymphocystisvirus*, *Megalocytivirus*, *Ranavirus*, *Chloriridovirus*, *Daphniairidovirus* and *Decapodiridovirus*, and *Iridovirus* [2,3,4]. Megalocytiviruses are an emerging group of closely related dsDNA viruses that cause systemic infections in a wide variety of wild and cultured fresh and saltwater fishes. Megalocytivirus outbreaks are of considerable economic importance in aquaculture, as epizootics can result in moderate fish loss or mass mortality events of cultured fishes. The infection is characterized by obvious hypertrophy of infected cells [3,5]. *Megalocytivirus* is the newest genus within the family, mainly including Red Sea bream iridovirus (RSIV) [6], infectious spleen and kidney necrosis virus (ISKNV) [7], rock bream iridovirus (RBIV) [8], Turbot reddish body iridovirus (TRBIV), and scale drop disease virus (SDDV) [9]. Based on sequence analysis and serological studies, all megalocytiviruses isolated to date appear to be strains of two viral species. Phylogenetic analysis indicates the presence of two clusters, one large cluster composed of RSIV, ISKNV, TRBIV, and other highly similar viruses, and a second, more distant, cluster comprised of a single isolate, SDDV. ISKNV, first reported in mandarin fish (*Siniperca chuatsi*), is a pathogen that causes high mortality in mandarin fish. It belongs to *Megalocytivirus*, in the *Iridoviridae* family [5,7,10]. ISKNV infection of mandarin fish causes cellular hypertrophy in the spleen, kidney, cranial connective tissue, and endocardium [7,10]. The ISKNV genome (GenBank accession number AF371960) was sequenced in 2001 and is highly methylated [7]. Recent research has found that many fish are infected by a virus that causes clinical signs similar to ISKNV infection. These viruses are often called infectious spleen and kidney necrosis virus-like (ISKNV-like) viruses. ISKNV-like viruses can infect over 60 other species of marine and freshwater fish, such as largemouth bass (*Micropterus salmoides*) [10,11,12,13]. Because ISKNV-like viruses cause significant economic losses, the isolation and identification of ISKNV-like virus can contribute to the prediction and prevention of viral disease outbreak [12].

Largemouth bass (*M. salmoides*) is an important freshwater aquaculture species and has been widely cultured in China in recent years. According to statistics, the production of largemouth bass in China reached 0.67 million tons in 2021 [14]. However, with the increase in breeding density, disease-related challenges are becoming increasingly serious. Especially the emergence of viral pathogens has brought huge economic losses to largemouth bass breeding. Several viruses have been reported to infect largemouth bass and cause high mortality, such as largemouth bass virus [15], largemouth bass ulcer syndrome virus [16], and micropterus salmoides rhabdovirus [17].

In 2017, we tested samples of diseased largemouth bass from Zunyi, Guizhou Province, China, and found that many samples were detected with megalocytiviruses. We then isolated the virus and identified it as an ISKNV-like virus (designated as ISKNV-ZY). The features and pathogenicity of ISKNV-ZY were also investigated. The present study provides information for conducting further research on the interaction between largemouth bass and ISKNV.

## 2. Materials and Methods

### 2.1. Collection and Processing of Sick Fish Samples

Diseased largemouth bass with an average length of 15 cm were obtained from an aquaculture farm in Zunyi City, Guizhou Province, China in 2017. The fish were quickly dissected, and tissues such as the liver, spleen, and kidney were collected and frozen in liquid nitrogen and stored at −80 °C.

### 2.2. Fish and Cell

Mandarin fish brain (MFB) cells were provided by Dongzhuo Zhang. Cells were constructed from the brain tissue of mandarin fish and have been cultured for more than 100 generations. Cells were serially passaged every four days and grown at 28 °C in Leibovitz’s L-15 medium (Gibco, Grand Island, NY, USA) supplemented with 15% fetal bovine serum (Gibco) for further use.

Healthy largemouth basses averaging 7.4 ± 0.4 cm in length and weighing 11 ± 0.3 g were obtained from the fishery in Guangzhou, Guangdong province, China. They were stored in a recirculating freshwater system at 24–28 °C and fed twice daily for two weeks. Then three groupers were randomly selected to detect whether the fish was infected with bacteria or viruses.

### 2.3. Virus Detection

The tissues of diseased fish were isolated for DNA extraction and PCR analyses. Genomic DNA was extracted using the TIANamp Genomic DNA Kit (Tiangen Biotech, Beijing, China). Megalocytivirus major capsid protein (MCP)-specific primers, MCP-F1 (5′-ATGTCTGCAATCTCAGGTGCAAACGTAAC-3′) and MCP-R1 (5′-TTACAGGATAGGGAAGCCTGCGGCGCCG-3′), were designed for PCR based on the sequence of MCP (GenBank accession number: ON743043.1). The expected result of PCR is 1362 bp. The PCR reaction conditions were as follows: a cycle of 95 °C for 8 min, 35 cycles of 94 °C for 45 s, 56 °C for 45 s, and 72 °C for 1 min, and a final extension step at 72 °C for 10 min. The PCR products were detected using 1% agarose gel electrophoresis.

### 2.4. Virus Isolation

The spleens of diseased largemouth bass were homogenized in sterile PBS and filtered through a 0.22 µm filter. The filtrate was freeze-thawed three times, and then incubated with MFB cells. Cytopathological effects (CPE) were observed using a microscope. Infected cells were collected as virus stocks. Viral titers were determined using the TCID_50_ assay.

### 2.5. Transmission Electron Microscopy Assay

MFB cells infected with ISKNV-ZY were collected, washed thrice with 0.1 M phosphate buffer (pH 7.4), and then fixed with 2.5% glutaraldehyde in 0.1 M phosphate buffer (pH 7.4) overnight at 4 °C, followed by post-fixation with 1% osmium tetroxide. Sections were double stained with uranyl acetate and lead citrate. Grids containing ultrathin sections were examined at 120 kV using a Talos L120 C transmission electron microscope (Thermo Fisher Scientific, Waltham, MA, USA). Micrographs were taken using a CDD camera.

### 2.6. Complete Genomic Analysis of ISKNV-ZY in Cell Culture

The complete genome of ISKNV-ZY was identified, and the remaining gaps were amplified using primers designed based on the assembled sequences and the reference genome close to the gaps. The gaps were amplified with LA Taq DNA polymerase (with GC buffer; TaKaRa, Kusatsu City, Japan), and the products were subjected to gel-based purification and cloned for sequencing. Open reading frames (ORFs) were predicted using the Geneious Prime 2021.2.1 software. The amino acid sequences of the core iridovirus genes DNA polymerase, MCP, ATPase, and ribonuclease III were aligned using CLUSTAL X 1.83. The phylogenetic trees were constructed using the Neighbor-Join method in the MEGA 7.0 software.

### 2.7. Experimental Infection

Thirty healthy largemouth basses were injected intraperitoneally with 10^4.5^ TCID_50_/mL of virus suspension (infection group). Sterile PBS was injected in the control group. Fish were placed in 300 L of artificial freshwater and maintained at 28 °C. Dead fish were recorded daily, and cumulative mortality was calculated based on the data collected until 14 days post-infection (p.i.).

### 2.8. Transcriptomic Analysis of Virus-Infected Largemouth Bass

The spleen tissues of 12 virus-infected largemouth bass were collected and randomly divided into three groups (infected groups at seven days p.i.). The spleen tissue of healthy largemouth bass was taken in the same way and divided into three groups (control groups). Total RNA was extracted, and the libraries were constructed and sequenced on the Illumina sequencing platform (HiSeqTM 2500). The functions of unigenes were annotated by aligning unigenes to orthologous clusters of NCBI Non-Redundant, SwissProt, and Eukaryotic Complete Genomes (KOG) databases using Blastx. DESeq2 was used to identify differentially expressed unigenes (DEGs). Gene Ontology (GO) enrichment and Kyoto Encyclopedia of Genes and Genomes (KEGG) pathway enrichment analysis of the DEGs were, respectively, performed using R based on the hypergeometric distribution.

### 2.9. Quantitative Real-Time Polymerase Chain Reaction (qRT-PCR) Analysis

Different DEGs were selected from different signaling pathways to verify the reliability of transcriptomic results. An Applied Biosystems QuantStudio 5 Real-Time PCR System (Thermo Fisher, Waltham, MA, USA) was used to perform qRT-PCR. The target gene primers are listed in Table 1. β-actin was used as the internal reference gene. The expression levels were calculated using the 2^−ΔΔCT^ method.

### 2.10. Statistical Analysis

GraphPad Prism (version 8.0.2) was used to perform statistical analysis. Data analysis results were shown as the mean ± standard error of the mean of three independent experiments. Statistically significant differences were evaluated using the *t*-test. *p*-values of * <0.05 and ** <0.01 were considered statistically significant.

## 3. Results

### 3.1. Pathological Signs of Diseased Fish

Compared with healthy largemouth bass, the diseased fish showed a loss of appetite, hypoxia, abnormal swimming, and a slightly yellowish abdomen. After dissection, we found that the spleen and kidneys were significantly enlarged, which was very similar to the symptoms after ISKNV infection. Accordingly, the tissues of the liver, spleen, and kidney were collected, DNA was extracted, and a PCR reaction was carried out with the MCP primers of ISKNV. The results of the gel electrophoresis showed that there was a band with a size of 1362 bp consistent with the expected size. The PCR products were sent to the company for sequencing, and the comparison of the sequencing results showed that it was identical to the MCP sequence of ISKNV. Therefore, the virus was named ISKNV-ZY.

### 3.2. Virus Isolation

**Figure 1:**
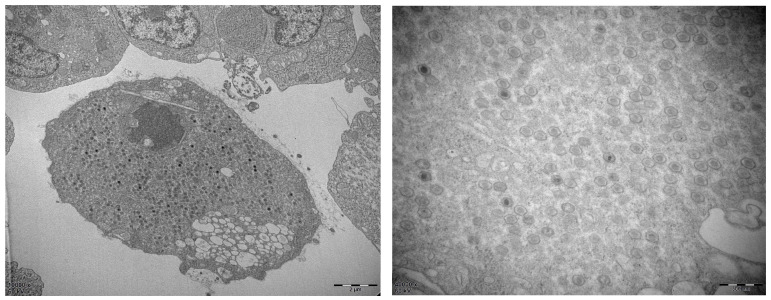
Viral particles examined using electron microscopy.

In order to better study the infection mechanism of the virus, we isolated and identified the new virus. First, we added the filtrate of the spleen tissue to the MFB cells. After three generations of blind passage, some cells were pathological. The infected cells were collected and frozen and thawed repeatedly as the original virus solution. The virus was added to the MFB cells again, and after four days, a few cells gradually became round and swollen. After seven days, significant CPE was visible. The virus-infected cells were collected, electron microscope sections were prepared, and the virus particles were observed using an electron microscope. As shown in Figure 1, a large number of hexagonal virus particles (approximately 131 nm in diameter) were observed in the cell cytoplasm.

### 3.3. Genome Sequencing and Functional Annotation

**Figure 2:**
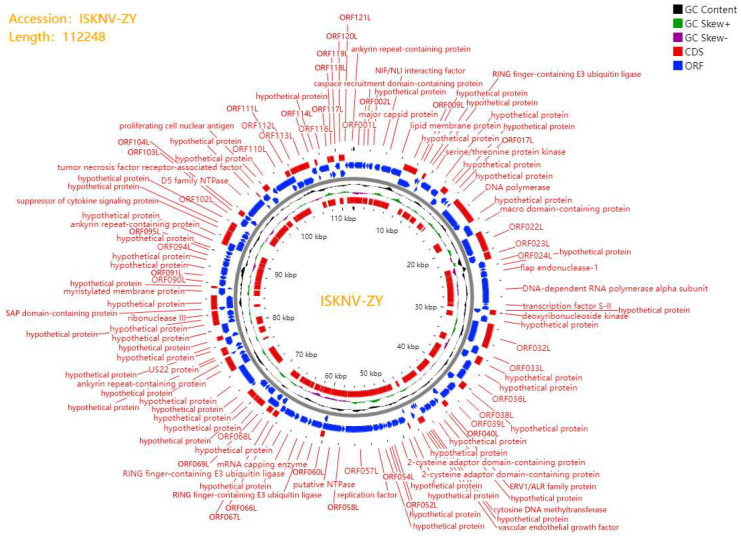
Infectious spleen and kidney necrosis virus (ISKNV)-ZY genome circle mapping using the Proksee online software (https://proksee.ca/, 7 January 2023). The orange portion denotes the predicted open reading frames (ORFs), with the arrow direction indicating the approximate size and transcription direction of the ORFs (https://www.ncbi.nlm.nih.gov/orffinder/, 7 January 2023). The blue part is the CD information subjected to a homology search using the NCBI BLASTp (https://blast.ncbi.nlm.nih.gov/Blastp, 7 January 2023) program. The circle diagram also shows the CG content and CG Skew distribution of the genome.

**Figure 3:**
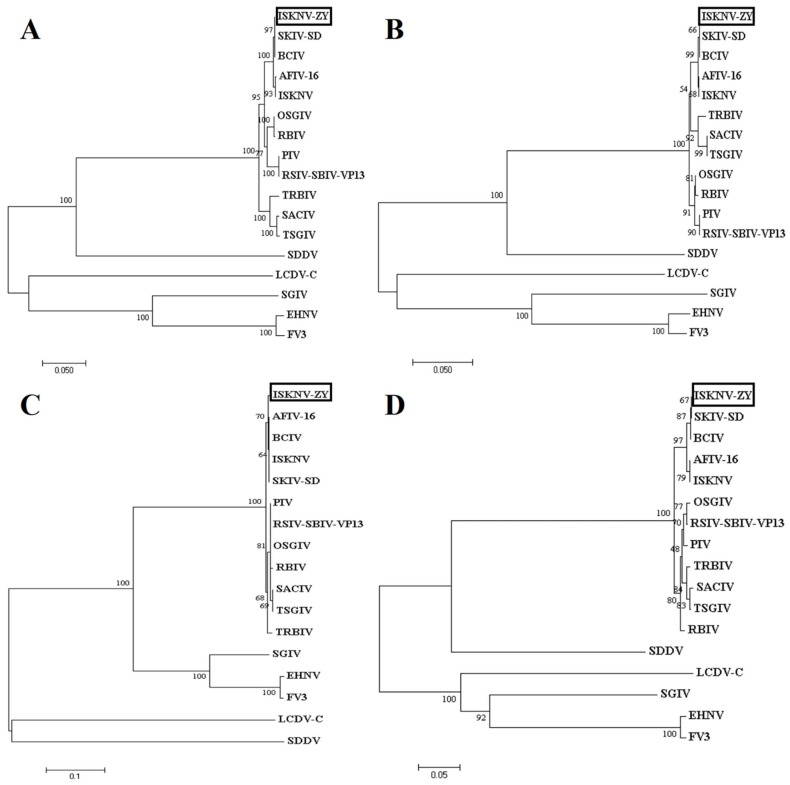
Phylogenetic relationship of infectious spleen and kidney necrosis virus (ISKNV)-ZY to other fish iridoviruses. Phylogenetic trees based on the amino acid sequences of DNA polymerase (**A**), Megalocytivirus major capsid protein (MCP) (**B**), ATPase (**C**), and ribonuclease III (**D**) were constructed using the MEGA software. All viral sequences are from the GenBank database (accession numbers: OSGIV, AY894343; ISKNV, NC_003494; RBIV, AY532606; BCIV, MN432490; RSIV-SBIV-VP13, ON740976; TRBIV, GQ273492; TSGIV, MG570132; SACIV, MG570131; SKIV-SD, MT986830; AFIV-16, MK689685; PIV, MK098185; SGIV, NC_006549; LCDV-C, NC_005902; EHNV, NC_028461; FV3, NC_005946; SDDV, KR139659).

**Figure 4:**
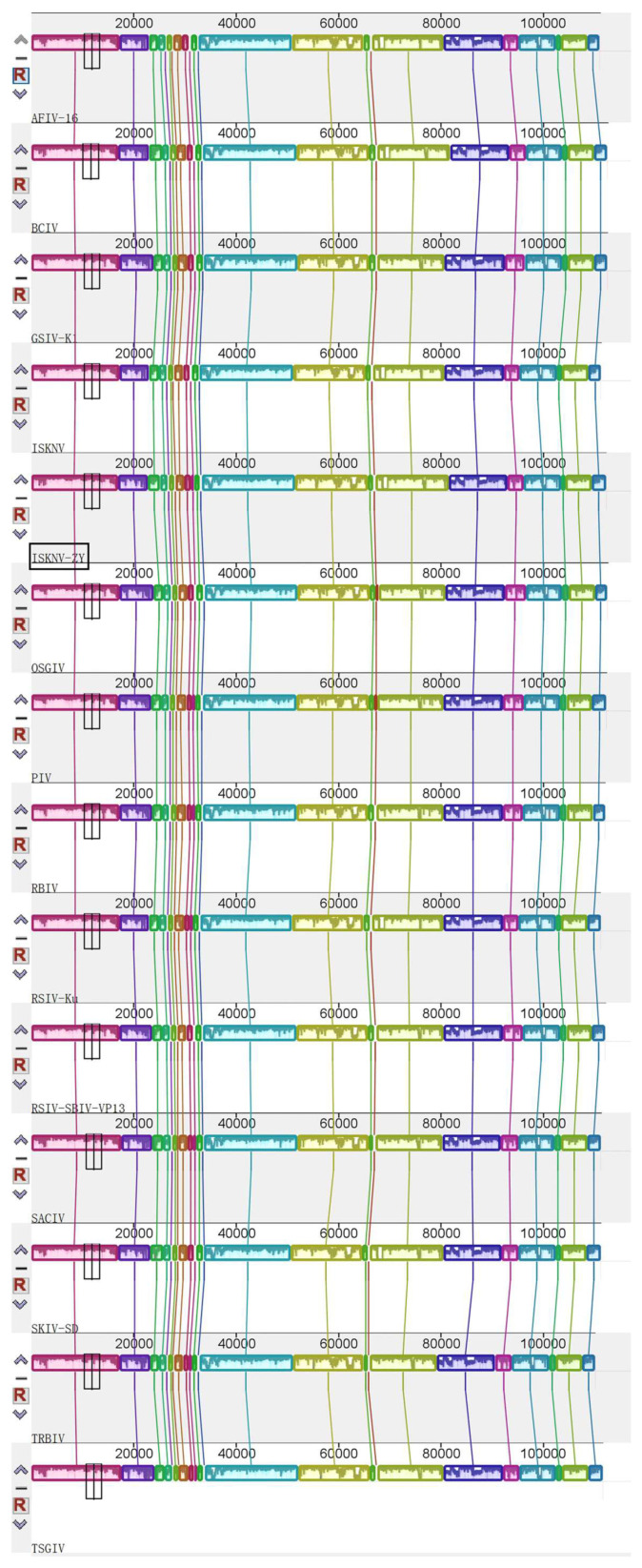
Genome collinearity between infectious spleen and kidney necrosis virus (ISKNV)-ZY and other *Megalocytiviruses*. Data were obtained on 8 October 2021 using the MAUVE software (http://darlinglab.org/mauve/mauve.html), which analyzes population evolutionary events through multiple sequence comparisons. Accession numbers of viral genomes are: OSGIV, AY894343; ISKNV, NC_003494; RBIV, AY532606; BCIV, MN432490; RSIV-Ku, KT781098; RSIV-SBIV-VP13, ON740976; TRBIV, GQ273492; TSGIV, MG570132; SACIV, MG570131; SKIV-SD, MT986830; GSIV-K1, KT804738; AFIV-16, MK689685 and PIV, MK098185.

The genome of ISKNV-ZY was sequenced on the Illumina Novaseq platform. The full length of ISKNV-ZY is 112,248 bp, and the GenBank accession number is OP009387. The GC content is 54.9%, which is consistent with the average GC content of megalocytivirus. A total of 122 ORFs were found in the ISKNV-ZY genome, encoding at least 120 amino acids, including 26 core conserved proteins (Figure 2). If the transmembrane amino acid transporter gene is assigned as the first ORF (counterclockwise), there are 47 ORFs in the clockwise direction and 75 ORFs in the counterclockwise direction. Among the 122 ORFs, 96 had 100% homology with the compared viral genes. Among the 122 ORFs, 81 had the highest homology with Banggai cardinalfish Iridovirus (BCIV), and other ORFs had a higher homology with ISKNV, Spotted knifejaw iridovirus Shangdong strain (SKIV-SD), and pompano Iridovirus (PIV). Phylogenetic trees were constructed using the MEGA7.0 software based on the protein sequences of the four core conserved proteins DNA polymerase, MCP, ATPase, and ribonuclease III of iridovirus, and the results showed that ISKNV-ZY was closely related to BCIV, ISKNV, and SKIV-SD (Figure 3). Both BCIV and SKIV-SD belong to the ISKNV-like isolates that were more related to ISKNV. The results of gene collinearity based on the physical location of the genome and protein similarity showed that the genome sequence of ISKNV-ZY was completely consistent with BCIV, ISKNV, SKIV-SD and several other genotypes (Figure 4). In summary, ISKNV-ZY should be an ISKNV-like virus.

### 3.4. Experimental Infection of Isolated Viruses in Largemouth Bass

**Figure 5:**
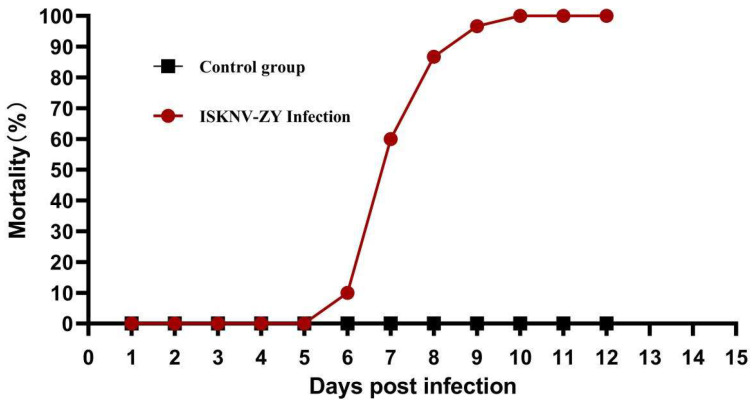
Pathogenicity of infectious spleen and kidney necrosis virus (ISKNV)-ZY in largemouth bass. Cumulative mortality of largemouth bass after challenge with ISKNV-ZY was recorded until day 12 post-infection.

The mortality of largemouth bass was recorded daily following viral infection. Largemouth basses began to die on the sixth day in the infected group, reached the peak of death on days 7–8, and all died on day 10 (Figure 5). The pathological features were the same as those of natural infection. No fish died in the control group. The spleens of the dead fish and the normal fish were obtained for PCR detection. The results showed that the bands of MCP were clearly detected in the dead fish, and the size was consistent with the expectation, while the control group did not. The PCR products were sent to the company for sequencing, and the comparison of the sequencing results showed that it was identical to the MCP sequence of ISKNV-ZY. The above results indicated that ISKNV-ZY can infect largemouth bass with high mortality rates.

### 3.5. Transcriptomic Analysis of Virus-Infected Largemouth Bass

**Figure 6:**
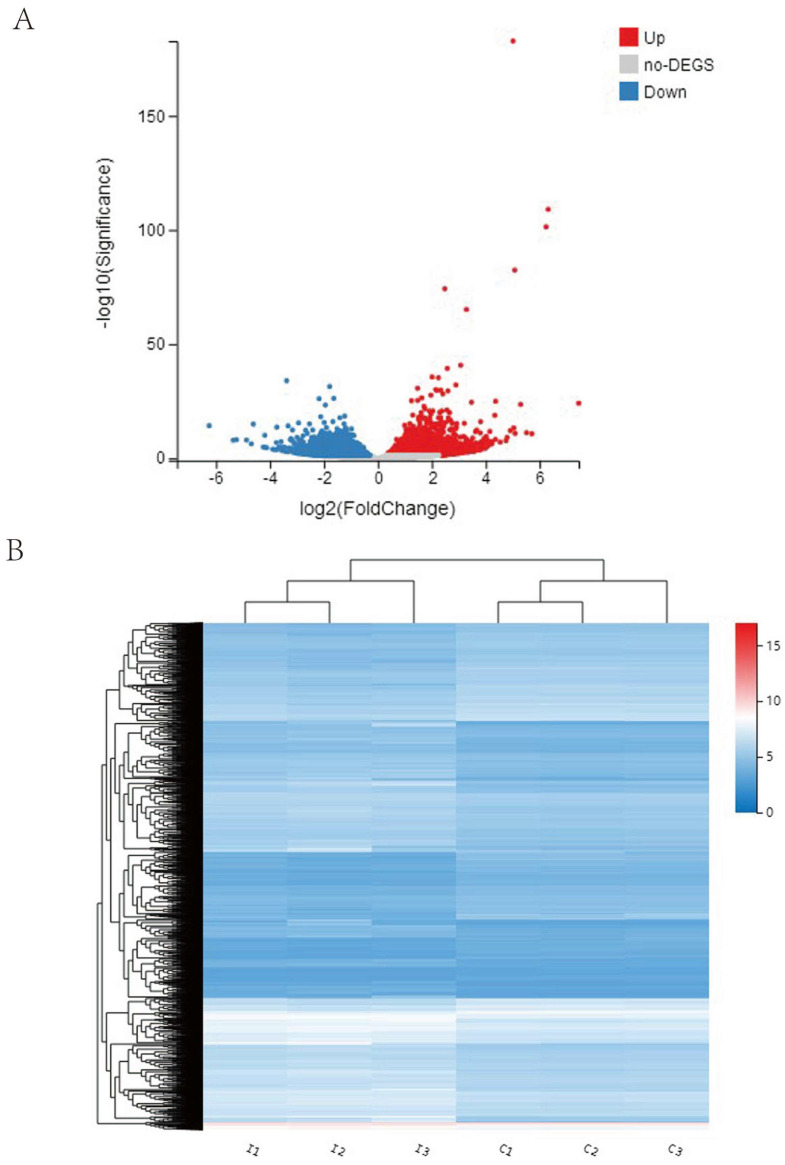

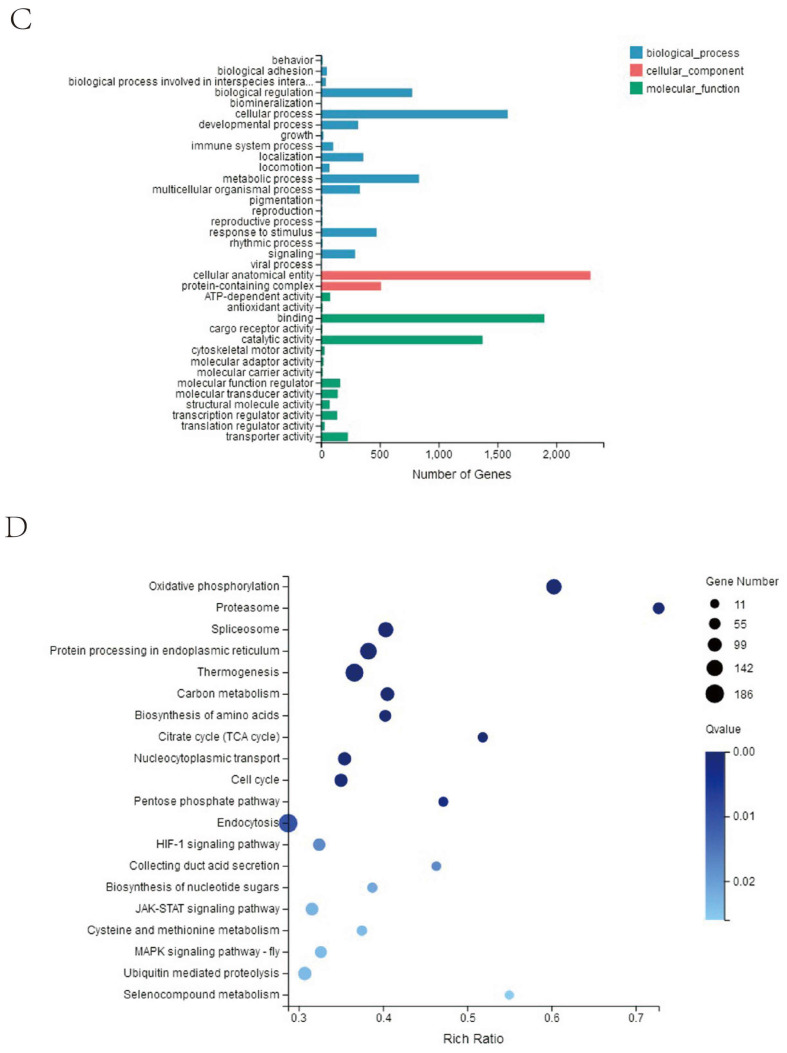
Differential gene expression, clustering, and functional enrichment analysis. (**A**) Volcano plot analysis of differentially expressed unigenes (DEGs). The horizontal ordinate represents the fold change in gene expression in the different experimental groups and the vertical ordinate represents the statistical significance of the change in gene expression. Red plots represent a significant enrichment of DEGs (*p* < 0.05). Green plots represent significant downregulation of DEGs (*p* < 0.05). (**B**) Hierarchical clustering analysis of total genes between the control and infectious spleen and kidney necrosis virus (ISKNV)-ZY-infected groups (fold change > 2, *p* < 0.05). The color in the heat map represents gene expression changes. (**C**) Gene Ontology (GO) terms in biological process, cellular component, and molecular function categories with enriched DEGs. (**D**) Top 20 enriched pathways for the total genes on Kyoto Encyclopedia of Genes and Genomes (KEGG) analysis.

Transcriptional analysis of the spleens from the moribund and healthy largemouth bass was subsequently performed. A total of six samples (three control samples and three ISKNV-ZY-infected samples) were measured using the DNBSEQ platform, and each sample produced an average of 6.44 G. The effective data volume of each sample was distributed in the range from 6.4 to 6.46 G, and the distribution of Q30 bases was 94.1–95.09%. The average alignment rate of samples compared to the genome was 93.62%, and the average alignment rate of the compared gene set was 80.08%. A total of 6254 DEGs, of which 3518 were upregulated and 2673 downregulated, were identified (Figure 6A). Differential gene expression clustering heat map results showed that the control and ISKNV-ZY-infected groups were divided into two clusters according to the dendritic branches (Figure 6B). GO enrichment analysis on the DEGs was performed, and the top genes in each category are shown in Figure 6C. The biological processes were enriched in cellular process, metabolic process, biological process, and response to stimulus. The most enriched cellular components were cellular anatomical entities and protein-containing complexes. The largest subcategory of molecular function was binding. The results of the KEGG enrichment analysis showed that the DEGs were mainly enriched in endocytosis, thermogenesis, protein processing in the endoplasmic reticulum, oxidative phosphorylation, the JAK-STAT signaling pathway, MAPK signaling pathway, and ubiquitin-mediated proteolysis (Figure 6D).

### 3.6. DEG Validation Using qRT-PCR

**Figure 7:**
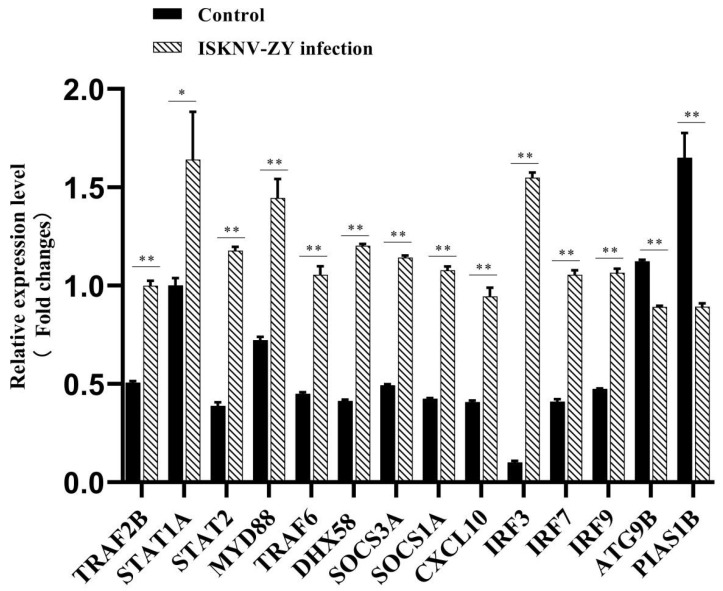
Relative expression levels of 14 genes in largemouth bass infected with infectious spleen and kidney necrosis virus (ISKNV)-ZY. The fold change in the expression of the 14 selected upregulated and downregulated differentially expressed unigenes (DEGs) was validated using qRT-PCR upon ISKNV-ZY infection. Data are expressed as log2 (fold change) relative to β-actin. *p*-values of * <0.05 and ** <0.01 were considered statistically significant.

Fourteen genes were randomly selected from different pathways to verify differential gene expression. These pathways included the RIG-I-like receptor signaling pathway, cytokine-cell Factor-receptor interaction, the Jak-STAT signaling pathway, and autophagy pathway. The results showed that the expression patterns of the candidate genes were consistent with the transcriptome profile analyses, suggesting that the transcriptome data were reliable (Figure 7).

## 4. Discussion

The megalocytivirus, one of the pathogens responsible for the high mortality in a variety of freshwater and marine fish, has brought huge economic losses to the aquaculture industry worldwide [3]. RSIV was the first discovered megalocytivirus and one of the best characterized [5,6]. The first outbreak of megalocytiviral disease was recorded in cultured red sea bream (*Pagrus major*) in Japan in 1990 and was designated the red sea bream iridovirus disease (RSIVD). Following infection fish became lethargic and exhibited severe anemia, petechiae of the gills, and enlargement of the spleen [3]. Subsequently, some apparently similar viruses, such as ISKNV [7], Taiwan grouper iridovirus [18], TRBIV [9], RBIV [19], and dwarf gourami iridovirus [20] were isolated. At present, these viruses can be tentatively classified into three species represented by RSIV, ISKNV, and TRBIV [3,5]. The largemouth bass is an important farmed fish in China with high economic value. However, with the influence of breeding density and breeding environment, disease-related problems are becoming increasingly serious [14]. When we conducted an epidemiological investigation on largemouth bass cultured in Zunyi, Guizhou Province, China, we found that many largemouth basses had death signs similar to those of megalocytivirus infection. Therefore, we quickly shipped the samples back to the laboratory for identification. Clinical symptoms and PCR results indicated that the main cause of death of largemouth bass was infection with megalocytivirus. In the present study, the virus was isolated and identified and its immune mechanism after infection was studied.

After grinding and filtering, the spleen tissue of diseased largemouth bass was added to the MFB cells, which were blindly passed for three generations, and the virus infection solution was successfully obtained. Using electron microscopy, we observed that the viral particles were hexagonal with a diameter of approximately 131 nm, very similar to those of megalocytivirus. Megalocytiviruses are large icosahedral DNA viruses measuring 120–200 nm in diameter [5,21]. The viral particles of ISKNV were also hexagonal with a diameter of approximately 150 nm. Under electron microscopy, the viral particles of SKIV-SD were approximately 140 nm in diameter. The average width of BCIV virus particles was 126 nm with a range from 122 to 130 nm.

The megalocytivirus genome is a linear dsDNA molecule that is circularly arranged and terminally redundant. Currently, the complete genome sequences of five megalocytiviruses including ISKNV (AF371960), RSIV-Ehime1 strain (BD143114, AB104413), RBIV(AY532606), OSGIV(AY894343), TRBIV (GQ273492), and large yellow croaker iridovirus (AY779031) have been reported [7,22,23,24,25]. Their genome length and GC content ranged from 110,104 to 112,636 bp, and from 53 to 55%, respectively. The sequence of the ISKNV genome is 111,362 bp with a GC content of 54.78% [7]. The full length of ISKNV-ZY is 112,248 bp, and the GC content is 54.9%, which is consistent with ISKNV. ISKNV contained 124 potential ORFs with coding capacities ranging from 40 to 1208 amino acids, of which 35 show significant homology to functionally characterized proteins from other species [7]. RSIV Ehime-1 encodes 116 ORFs, of which approximately 30 genes are common to all iridescent viruses and 86 are specific to RSIV [26]. ISKNV-ZY encodes 122 ORFs, of which 81 have the highest homology with BCIV, and the remaining have higher homology with ISKNV, SKIV-SD, and PIV. The MCP and ATPase genes were frequently used for phylogenetic analysis of multiple Megalocytivirus isolates [3]. Analysis of the MCP and ATPase genes showed that the genus *Megalocytivirus* can be divided into three clusters represented by RSIV, ISKNV, and TRBIV [22,27,28,29,30]. Based on the protein sequences of the four core conserved proteins MCP, ATPase, DNA polymerase, and ribonuclease III of iridovirus, the results of the phylogenetic tree showed that ISKNV-ZY was closely related to BCIV, ISKNV, and SKIV-SD. Therefore, we speculate that ISKNV-ZY, BCIV, and SKIV-SD are of the same type, and all belong to the ISKNV-like megalocytivirus [12]. ISKNV-ZY was more related to ISKNV, and it should be considered a variant of ISKNV. Previous studies also found two clusters were observed in the ISKNV-like viruses from the infected fish [12]. Cluster I included the ISKNV-like isolates that were more related to ISKNV, and they should be considered variants of ISKNV. Cluster II consisted of OSGIV from orange-spotted grouper and RBIV, which were different from ISKNV [12]. The results of gene collinearity further show that the genome sequence of ISKNV-ZY was completely consistent with BCIV, ISKNV, SKIV-SD, and several other genotypes.

To further explore the pathogenicity of the virus isolates, we conducted infection experiments. The results of the study showed that largemouth bass began to die on the sixth day after virus infection, reached a death peak on days 7–8, and all fish died after 10 days. The symptoms of death were similar to those of natural infection, and the spleen was enlarged. PCR results showed that a significant virus amount was detected in the spleen. The above results indicated that the newly isolated virus had higher pathogenicity.

Transcriptome sequencing technology is a technology developed in recent years that can provide important insights into the immune response of fish to viral infection. In a previous study, the transcriptomics of spleen tissues after ISKNV infection in mandarin fish were studied. The results showed that the DEGs were annotated to 52 KEGG pathways, including five immune-related pathways, which were the leukocyte transepithelial migration pathway, complement and coagulation cascade pathway, chemokine signal pathway, Fc gamma R-mediated phagocytosis pathway, and natural killer cell-mediated cytotoxicity pathway [31]. Transcriptome analysis of differential functional gene expression in largemouth bass after challenge with *Nocardia seriolae* showed that the differential expression of the immune-related genes was identified from 13 pathways, mainly including cytokines and their receptors, the Toll-like receptor signaling (TLR) pathway, and the T cell receptor signaling pathway. *N. seriolae* infection also affected genes significantly associated with transcriptional regulation, including NF-κB signaling and JAK-STAT signaling [32]. To further reveal the response mechanism of largemouth bass to emerging viral infection, we performed transcriptomic analysis before and after viral infection. The results of the KEGG enrichment analysis showed that the DEGs were mainly enriched in endocytosis, thermogenesis, protein processing in the endoplasmic reticulum, oxidative phosphorylation, the JAK-STAT signaling pathway, MAPK signaling pathway, and ubiquitin-mediated proteolysis. The above results show that the immune signaling pathways mediated by the same virus differ in different fish, and that the immune signaling pathways mediated by different pathogens in the same fish are also different.

In fish, pattern recognition receptors, such as TLRs, RIG-I-like receptors, NOD-like receptors, and C-type lectin receptors [33,34,35], recognize pathogen-associated molecular patterns to defend against pathogen invasion and activate immune responses through signaling pathways. In this study, a total of 12 gene transcripts were found to be upregulated and two gene transcripts were found to be downregulated. The expression of RIG-I-like receptor-related genes, such as dhx58, IRF3, IRF7, TRAF6, and STAT1A in our results, is consistent with that in the study on largemouth bass in the spleen after immersion challenge with *N. seriolae* [32]. In a previous study, it was found that different cytokines and cytokine receptor families are upregulated in the cytokine–cytokine receptor interaction signaling pathways after *N. seriolae* infection. We analyzed the expression of CXCL10, and the results were consistent with those after *N. seriolae* infection [32]. These data indicate that the cytokine–cytokine receptor interaction may be an important antibacterial and antiviral mechanism.

In the present study, an ISKNV-like virus (named ISKNV-ZY) from cultured largemouth bass was isolated and characterized. The whole genome of ISKNV-ZY contained 112,248 bp and 122 open reading frames. The transcriptomic analysis conducted in this study provides resources for further exploring the immune response mechanism of largemouth bass to ISKNV and molecular data and targets for the genetic improvement of largemouth bass against viral diseases.

## Figures and Tables

**Table 1:** Target gene primers used in quantitative real-time PCR analysis.

Primers	Sequence (5′–3′)	GenBank Accession Number
β-actin-RT-F	CCACCACAGCCGAGAGGGAA	MH018565.1
β-actin-RT-R	TCATGGTGGATGGGGCCAGG	
TRAF2B-RT-F	AACAACAGGGAGCACATT	XM_038734170.1
TRAF2B-RT-R	GCCAGCCAGTTTAGCC	
STAT1A-RT-F	AACTGCTCGGAGCCAAAG	XM_038715521.1
STAT1A-RT-R	CAGCCAGAAGGGAAACG	
STAT2-RT-F	GGCCGACCTAAATAAGC	XM_038696682.1
STAT2-RT-R	AAGAAACTCCCGCACC	
MYD88-RT-F	TGCCTTCATCTGCTACTGC	XM_038728827.1
MYD88-RT-R	CACCATCCGCTTACACCT	
TRAF6-RT-F	AATAAGGGCAGTTTGGAG	XM_038700569.1
TRAF6-RT-R	GAAGGGCTGAGTAGGGA	
DHX58-RT-F	GGGCTGGCAATGGTA	XM_038704577.1
DHX58-RT-R	GCTGCTGGGCAATCTC	
SOCS3A-RT-F	CCTCACCGTTACAAGACC	XM_038701469.1
SOCS3A-RT-R	CCTCGGAAGCCAGCAT	
SOCS1A-RT-F	ATGGTAGCCGAAAGCACA	XM_038696259.1
SOCS1A-RT-R	AGGGACGGACACCAATCT	
CXCL10-RT-F	GCTATTGCCTGAACCC	XM_038699228.1
CXCL10-RT-R	TGCTAGTGCTGCGTGT	
IRF3-RT-F	CTGCGAGCACAGATTGAC	XM_038735464.1
IRF3-RT-R	GCCCACGCCTTAAAGAT	
IRF7-RT-F	TGTAGCACCCGAGAACC	XM_038706685.1
IRF7-RT-R	GGAATGTGCCCTTTAGC	
ATG9B-RT-F	AAAAGGTGCAACACGATG	XM_038717820.1
ATG9B-RT-R	GTCCTGACGCAGTTAGAAT	
PIAS1B-RT-F	CCCCATCAACATAACCTC	XM_038706189.1
PIAS1B-RT-R	AATTCCTTTAGCCCGTAG	
IRF9-RT-F	GAAGCTGACGGATGGGAGT	XM_038732190.1
IRF9-RT-R	CGAGGCGGTAGACCTTGTAG	

## Data Availability

The datasets generated for this study are available on request to the corresponding author.
